# Modulators of actin-myosin dissociation: basis for muscle type functional differences during fatigue

**DOI:** 10.1152/ajpcell.00023.2017

**Published:** 2017-09-20

**Authors:** Christina Karatzaferi, Nancy Adamek, Michael A. Geeves

**Affiliations:** ^1^Muscle Physiology and Mechanics Group, DPESS, University of Thessaly, Trikala, Greece;; ^2^Experimental Myology and Integrative Physiology Cluster, FSHS, University of St Mark and St John, Plymouth, United Kingdom; and; ^3^School of Biosciences, University of Kent, Kent, United Kingdom

**Keywords:** myosin kinetics, cross-bridge cycle, temperature, muscle fatigue

## Abstract

The muscle types present with variable fatigue tolerance, in part due to the myosin isoform expressed. However, the critical steps that define “fatigability” in vivo of fast vs. slow myosin isoforms, at the molecular level, are not yet fully understood. We examined the modulation of the ATP-induced myosin subfragment 1 (S1) dissociation from pyrene-actin by inorganic phosphate (P_i_), pH, and temperature using a specially modified stopped-flow system that allowed fast kinetics measurements at physiological temperature. We contrasted the properties of rabbit psoas (fast) and bovine masseter (slow) myosins (obtained from samples collected from New Zealand rabbits and from a licensed abattoir, respectively, according to institutional and national ethics permits). To identify ATP cycling biochemical intermediates, we assessed ATP binding to a preequilibrated mixture of actomyosin and variable [ADP], pH (pH 7 vs. pH 6.2), and P_i_ (zero, 15, or 30 added mM P_i_) in a range of temperatures (5 to 45°C). Temperature and pH variations had little, if any, effect on the ADP dissociation constant (*K*_ADP_) for fast S1, but for slow S1, *K*_ADP_ was weakened with increasing temperature or low pH. In the absence of ADP, the dissociation constant for phosphate (*K*_Pi_) was weakened with increasing temperature for fast S1. In the presence of ADP, myosin type differences were revealed at the apparent phosphate affinity, depending on pH and temperature. Overall, the newly revealed kinetic differences between myosin types could help explain the in vivo observed muscle type functional differences at rest and during fatigue.

myosin ii exists in multiple isoforms (49), with slow muscles expressing myosin heavy chain type 1 (MyHC-1 also known as MyHC-β) and fast muscles expressing one or more of myosin heavy chain type 2 myosins (MyHC-2a, 2b, or 2x). Contraction depends directly on the interaction of myosin II multiheaded filaments, with filamentous “tracks” of actin, arranged within the sarcomeres, the “functional units” of muscle (28, 47). Eventually, whole muscle force output depends on the number of myosin cross-bridges interacting “strongly” or “weakly” with actin, while the velocity of contraction depends on the rate at which myosin detaches from actin at the end of the working stroke (11).

The study of kinetics of the actomyosin (A.M) interaction cycle identifies clear intermediate steps (for a review see Ref. 5). Such studies have revealed that slow skeletal myosin heavy chain isoforms (MyHC 1) have distinct properties from fast isoforms (MyHC 2s), e.g., regarding ATPase activity and the rate and equilibrium constants of the various biochemical steps, which are expected to dictate their different mechanical properties. Thus, efficiency of actin-induced ADP displacement from myosin [the ratio of the ADP dissociation constant for A.M (*K*_ADP_) over the ADP dissociation constant for myosin (*K*_D_)], and strain sensitivity (dependence on external mechanical load) can differ substantially between fast and slow myosins (5, 22), with slow myosins binding ADP tightly and releasing it at a slower rate than fast myosins. Consequently, ADP release is considered the rate limiting step for the maximum contraction velocity of slow muscles (29, 44), at least at the temperatures where fibers or myosin solutions are usually studied (10 to 22°C).

The coupling of biochemical steps with mechanical events has, however, not been fully elucidated (22), while the “laws” governing how ensembles of myosins integrate within the organized sarcomere (18, 19, 40) are not yet fully defined; this can be attributed partly to lack of physiologically relevant experimental evidence at the molecular level. This is especially true on the question of muscle fatigue, a complex multifaceted phenomenon.

At the organismal level, fatigue has a large heterogeneity of research outcomes (6) depending on the type, duration, and intensity of muscular activity employed (8, 10), the muscle composition studied (24), and health status (30), etc. In terms of intramuscular biochemical changes, the degree of acidosis observed depends on the rate and extent to which anaerobic glycolysis is relied upon; which in turn is dependent on the fiber type and type of activity (i.e., more in “supramaximal”/sprint type work, more in ischemia) as well as the presence and activity of lactate and proton transporters (see e.g., Ref. 32). Brief very intense voluntary exercise has been shown, in mixed muscle, to lower pH from 7.1 to 6.4 (7, 8, 27, 31, 48) and to disturb the ATP and phosphocreatine levels, notably in fast, type II, fibers to near depletion (34, 35). Based on NMR data, ADP levels are calculated to rise to 200 µM (21) as, in healthy muscle, they are well buffered by the adenylate kinase and AMP deaminase reactions (26). Still, small variations in [ADP] can significantly affect the sarcoplasmic reticulum’s function (39) and may help in maintaining tension economy (37). The drop in pH affects not only calcium sensitivity (20) but also the effect of accumulated inorganic phosphate (P_i_), which can reach 20–30 mM in exercising muscle (2, 38), with its di-protonated form considered to inhibit force (for a review see Ref. 2). At the myofibrillar level, changes in muscle mechanics during fatigue could be related to either reduction of energy substrates [e.g., causing localized ATP minima (34, 35)] and/or accumulation of ATP hydrolysis by-products (e.g., Refs. 14, 33, 37, 45, 53). This is because the interaction of myosin with actin (actomyosin) is a multisubstrate and multistep reaction, i.e., not only fueled by ATP hydrolysis but also modulated by ATP hydrolysis by-products (ADP, P_i_, H^+^) and other prevailing intracellular conditions (12). Thus, for the purposes of this work, fatigue is considered in the context of factors influencing the actomyosin cycle in a way to cause slowing of the cycle and/or weaker actomyosin interactions.

Overall, investigations ranging from whole body exercise (8, 30), to intact small muscles or fibers (59), to skinned fibers (13, 14, 17, 33, 37, 46), or myofibrils (53), and few isolated molecule approaches (e.g., Ref. 16) have provided strong evidence that the accumulation of inorganic phosphate (P_i_) and of hydrogen ions can contribute to, if not cause, peripheral muscle fatigue. Still, their exact impact, especially at physiological in vivo conditions, has attracted much debate (e.g., Ref. 58). This is further complicated by muscle type differences (fast vs. slow) in energetics, myosin ATPase, and mechanical performance (9, 49, 50), which can be linked to a great degree to inherent properties of the myosin II isoform expressed.

Our understanding of fatigue effects is further complicated by muscle type differences (fast vs. slow) in energetics, myosin ATPase, and mechanical performance (9, 49, 50), which can be linked to a great degree to inherent properties of the myosin II isoform expressed. The steps that control the detachment of the myosin cross-bridge at the end of the working stroke from actin are rapid and are thought to limit the shortening velocity, a key parameter of muscle function. Temperature predictions from kinetic studies of actomyosin in solution (44) suggest that the rate of ADP release may limit unloaded velocity for both fast and slow myosin isoforms. It can be hypothesized that such an ADP effect could be aggravated by the presence of hydrogen ions and inorganic phosphate, as in fatigue, but it is not known if this is the case and what would be the role of the myosin type.

Moreover, a parameter not often considered is temperature. In vivo mammalian muscle temperature ranges from 32 to >40°C, while in severe fatigue, pH drops and inorganic phosphate (P_i_) accumulates (23) concomitantly. A number of in vitro fiber studies at higher temperatures have challenged long-held views about the individual role of the key “fatigue” metabolites on mechanics [e.g., less of an effect of pH (36, 45, 59) or P_i_ on force (13, 14, 17, 33)]. Thus, it appears that employing temperature modulations in the in vitro experimentation is necessary to tease out physiological synergies [e.g., a synergism of myosin light chain phosphorylation with low pH and high [P_i_] became evident only at a high temperature (36)], if one wants to realistically link muscle function in vivo to actomyosin interaction molecular events studied in vitro. This necessitates molecular experimentation that mimics physiology to the degree possible.

Therefore the purpose of this research was to study the fast kinetics of ATP-induced dissociation of A.M with and without ADP using the stopped flow. We examined the interplay of “fatigue” factors, e.g., low pH and high inorganic phosphate (P_i_), with myosin type, on ATP-induced dissociation of A.M. Taking advantage of recent methodological advancements, we studied, for the first time, the ATP-induced dissociation of fast and slow S1 from actin in temperatures ranging from 5 to 45°C to reveal critical myosin type and/or temperature dependencies of these processes.

## MATERIALS AND METHODS

### Ethics Statement

Muscle tissue was obtained post mortem from animals treated as recommended by national and local guidelines using protocols approved under the UK Animal (Scientific Procedures) Act (1986). Fast skeletal muscle came from the psoas muscle of New Zealand rabbits and slow skeletal muscle came from bovine masseter.

### Protein Preparation

Myosin was prepared from the rabbit psoas (for fast MyHC-II) and the bovine masseter muscle (for slow MyHC-I) according to Margossian and Lowey (41), and was subsequently digested to subfragment 1 (S1) with chymotrypsin as described by Weeds and Taylor (57) which removes the regulatory light chain region. These two muscle types yield essentially pure MyHC isoform (e.g., Refs. 1, 25) for rabbit psoas (isoform 2X) and (55) for bovine masseter (isoform 1), a result confirmed in routine SDS-PAGE by us and others and by the expected value of *K*_ADP_ which is characteristic of a pure MyHC isoform (as indicated, e.g., in Ref. 4).

Actin was prepared from rabbit muscle as described by Spudich & Watt (52) and labeled with pyrene iodoacetamide to give pyrene-labeled actin as described by Criddle et al. (15). Protein stocks of S1 and of pyrene-labeled actin were stored at 4°C and were used for up to 2 wk. In the text herein, reference to actin implies pyrene-labeled actin.

### Experimental Buffers

The main buffer contained 20 mM cacodylate (adjusted at pH 7.0 or pH 6.2), 100 mM KCl, 5 mM MgCl_2_, and 1 mM NaN_3_; when phosphate was present in the buffer, the ionic strength was adjusted accordingly to a final ionic strength of 170 mM. Concentrations (whether of proteins or buffer constituents) given in the text and figure legends refer to the concentration after mixing 1:1 in the stopped flow (unless stated otherwise).

### Experimental Equipment, Procedures, and Analysis

Stopped-flow experiments were performed essentially as described previously (4) using a HiTech Scientific SF-61DX2 stopped-flow system; 4–5 transients were acquired for each ATP transient (Kinetic Studio suite). The dead time of the equipment was 0.002 s. A wide temperature range (5–45°C) for measurements was available because of a new adaptation of the standard stopped-flow machine [see Ref. 56). Briefly, the samples were held at room temperature (20°C) and then passed through a heat exchanger, at the temperature required for the measurement, just before entering the mixing chamber/observation chamber of the stopped flow. The samples were only exposed to the temperature of the measurement for a few seconds, thus allowing measurements of proteins under conditions where they are not usually stable for long.

The ATP-induced dissociation rate of actin.S1 was measured in the stopped flow by mixing a fixed concentration of pyr.actin.S1 complex (end concentration 0.25 µM) with excess ATP and monitoring fluorescence transients from the pyrene-labeled actin [excitation at 365 nm, emission through a KV389 nm cutoff filter (Schott, Mainz, Germany)]. Details of the kinetic analysis are given under *Data Fitting and Interpretation Approach*.

**Fig. 1.**
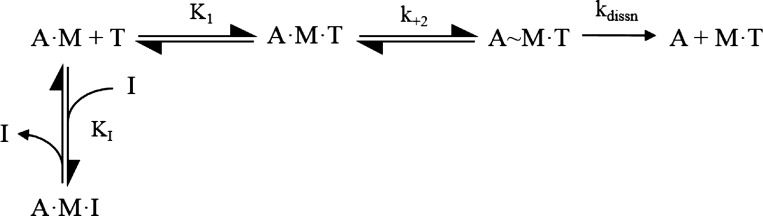
Model of ATP-induced dissociation of actin.S1. [Based on Millar and Geeves (42).]

In a similar process, ADP dissociation constant (*K*_ADP_), which defines ADP affinity for actin.S1, was measured by adding to the mixture ADP as a competitive inhibitor of ATP binding. In this case it is convenient to add the ADP to the ATP solution, i.e., 0.5 µM pyr.actin.S1 was mixed with 25 µM ATP with various concentrations of ADP present with the ATP (from 0 to 1200 µM). This approach assumes that ADP is in rapid equilibrium with the actin.S1 complex on the time scale of the ATP-induced dissociation reaction. This was ensured by using the low (25 µM) concentration of ATP. That this assumption holds was tested by repeating the measurement with ADP preincubated with actin.S1 and then mixing with ATP. The observed rate constants were identical in each case. Details of the data analysis are given below (see Fig. 1 on the competitive inhibitor approach and *Eqn 4*).

Phosphate dissociation constant (*K*_Pi_) was measured exactly as for the ADP dissociation constant except that the high concentrations of P_i_ used meant it was more convenient to have P_i_ present in the buffer in both syringes of the stopped flow. Details of the data analysis are given below (see Fig. 1 on the competitive inhibitor approach and *Eqns 5* and *6*).

ADP dissociation constant in the presence of phosphate (*K*_ADP+Pi_) was also measured using the same approach as for *K*_ADP_ but using buffers containing fixed amounts of inorganic phosphate, 30 mM in the case of psoas S1 and 15 mM with masseter S1. The different affinities of P_i_ for the two types of S1 required a different concentration of P_i_. Preliminary data indicated that P_i_ binding to psoas S1 was >10 mM and weaker than to masseter S1, by approximately a factor of 2. Since the limits of ionic strength precluded using saturation amounts of P_i_, we used a P_i_ concentration close to the range of *K*_Pi_ values.

Experiments were performed at two pH levels, 7 and 6.2 and in a range of temperatures. Care was taken to reverse the order of experiments to avoid the possibility of a time and “order” effect either with respect to pH or temperature.

### Data Fitting and Interpretation Approach

In the present study we focused our attention on the ATP-induced dissociation of actin.S1. This is the step that controls the detachment of the actomyosin cross-bridge at the end of the working stroke.

In Fig. 1, T = ATP; A = actin; M = myosin; I is an inhibitor, competitive with ATP for the nucleotide binding site. *K*_1_ defines the equilibrium constant for the formation of the A.M.T collision complex, which is followed by an almost irreversible isomerization of the complex to the ternary complex A~M.T with the rate constant of *k_+_*_2_. This is rapidly followed by dissociation of actin from the ternary complex. *K*_I_ is defined as a dissociation constant *k*_−I_/*k*_+I_. In the experiments presented here the inhibitor was either ADP or inorganic phosphate (P_i_).

**Fig. 2.**
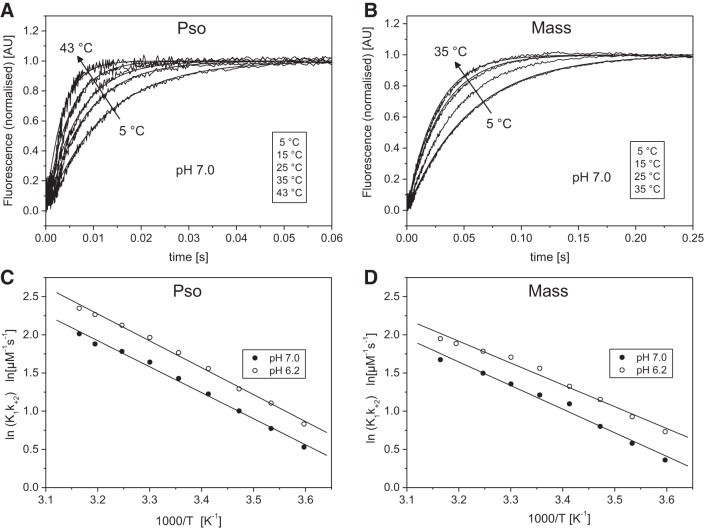
ATP-induced dissociation of S1 from actin, for fast (Pso) and slow (Mass) myosin isoform, at pH 7.0 and 6.2, in a range of temperatures. *A*: normalized transients observed when mixing 0.5 µM pyr-act.PsoS1 with 25 µM ATP in pH 7.0 buffer at different temperatures (selected transients are shown). The change in fluorescence was fitted to a single exponential equation (best fits superimposed), giving *k*_obs_ of 84.8, 136.2, 208.5, 296.9, and 374.4 s^−1^ for 5, 15, 25, 35, and 43°C, respectively. The amplitudes of the transients were relatively stable at 46% of total fluorescence change, with some loss observed at temperatures above 30°C. *B*: normalized transients observed when mixing 0.5 µM pyrAct.MassS1 with 25 µM ATP in pH 7.0 buffer at different temperatures (selected transients are shown). The change in fluorescence was fitted to a single exponential equation (best fits superimposed), giving observed rate constants of 26.7, 36.1, 46.7, and 64.9 s^−1^ for 5, 15, 25, and 35°C, respectively. The amplitudes of the transients were relatively stable at 40% of total fluorescence change, with some loss observed at temperatures above 30°C. *C*: Arrhenius plot of the *k*_obs_/[ATP] = *K*_1_*k*_+2_ of Pso at pH 7.0 and pH 6.2 (temperature range 5–43°C). The linear fits (best fits superimposed) gave slopes of −3.41 ± 0.10 and −3.52 ± 0.09 K for pH 7.0 and 6.2, respectively, from which the activation energies (*E*_a_) were calculated as 28.3 ± 0.8 and 29.3 ± 0.8 kJ/mol. *D*: Arrhenius plot of the *k*_obs_/[ATP] = *K*_1_*k*_+2_ of Mass at pH 7.0 and pH 6.2 (temperature range 5–43°C). The linear fits (best fits superimposed) gave slopes of −3.09 ± 0.17 and −2.86 ± 0.14 K for pH 7.0 and 6.2, respectively, from which the activation energies (*E*_a_) were calculated as 25.7 ± 1.4 and 23.8 ± 1.1 kJ/mol.

The reaction described in Fig. 1 was monitored through pyrene fluorescence changes which monitor the ATP-induced dissociation of actin from the complex (fluorescence increases by up to 70%.), specifically associated with step 2 of Fig. 1, (see results, Fig. 2*A*). Four to five transients were collected for each ATP concentration used then averaged before further analysis.

The averaged transients were fitted with a single (*Eqn 1*) or, if needed, a double exponential equation (*Eqn 2*):

(1)) $$F_{t}=\Delta F\cdot e^{(}{}^{-k_{obs}\cdot t)}+F_{\infty }$$

or

(2)) $$F_{t}=\Delta F_{\left(1\right)}\cdot e^{(}{}^{-k_{obs(1)}\cdot t)}+\Delta F_{\left(2\right)}\cdot e^{(}{}^{-k_{obs(2)}\cdot t)}+F_{\infty }$$

where *F*_t_ is the observed fluorescence at time *t*, *F*_∞_ is the fluorescence at the end of the transient (*t* = ∞) and Δ*F* is the total change of fluorescence observed. The observed rate constant (*k*_obs_) reflects the ATP induced dissociation rate of actin.S1 and is linearly dependent on [ATP], at the ATP concentrations used here. A plot of [ATP] vs. *k*_obs_ was used to derive the values of *K*_1_ and *k*_+2_ (using Origin software version 6.0), as defined in Fig. 1 and *Eqn 3*.

(3)) $$k_{obs}=K_{1}k_{+2}\left[ATP\right]$$

The presence of a competitive inhibitor to ATP binding (that does not induce actin.S1 dissociation) would appear to slow the rate of actin.S1 dissociation. If inhibitor binding is in rapid equilibrium with actin.S1, within the time scale of data acquisition, compared with the rate of ATP-induced dissociation of actin.S1 (i.e., *k*_+AD_ + [ADP]*k*_−AD_ >> *K*_1_*k*_+2_[ATP]), then

(4)) $$k_{obs}=K_{1}k_{+2}\left[ATP\right]/\left[1+([I]/K_{I})\right]$$

Then, plotting *k*_obs_ as a function of [I] will allow the *K*_I_ (in Fig. 1) to be defined. This approach was used to define the value of *K*_I_ for ADP (*K*_ADP_) and P_i_ (*K*_Pi_).

If both ADP and P_i_ are present in the same measurement, two scenarios are possible. If both compete for the same binding site then the effect of the two inhibitors is additive and the effect on *k*_obs_ can be predicted from the values of *K*_ADP_ and K_Pi_ measured independently.

(5)) $$k_{obs}=K_{1}k_{+2}\left[ATP\right]/[1+([ADP]/K_{ADP})+([P_{i}]/K_{Pi})]$$

where the measured *K*_I_ with variation of [ADP] and fixed [P_i_] is *K*_I_ = 1/*K*_ADP_ + [P_i_]/*K*_Pi._

If both, however, bind into the ATP pocket at the same time to create the complex A.M.ADP.Pi, then the above relationship will not hold and P_i_ will alter the affinity of A.M for ADP.

The apparent affinity of ADP for actin.S1 (*K*_ADP+Pi_) was measured for several concentrations of P_i_ and the dissociation constant of P_i_ was then calculated according to the following relationship and compared with the value of *K*_PI._

(6)) $$K_{\text{Pi app}}=\left[P_{i}\right]/\left(K_{\text{ADP+Pi}}/K_{\text{ADP}}-1\right)$$

### ADP Release Rate Constant

Two types of myosins were studied which are known to differ in their dissociation constant for nucleotides (5, 51). The rate constant for the release of ADP (*k*_−ADP_) is relatively slow for masseter S1 and can easily be measured in an ADP displacement experiment. This step is very fast for a fast muscle isoform and too fast to measure by current equipment. Briefly, actin.^Mass^S1 saturated with 75 µM of ADP (A.M.D complex) was mixed with a large excess of ATP (8 mM) in the stopped flow. Then the *k*_obs_ values, fitted to a single exponential equation (*Eqn 1*) defined the rate constant by which ADP is released by the ternary A.M.D complex (*k*_−AD_).

The data presented in the figures are the values for the individual experiment displayed, while the data values presented in Table 1 are averaged values for *n* = independent day measurements.

**Table 1.** Average values of kinetic parameters describing the ATP-induced dissociation rate of actin.S1 for psoas and masseter myosin, in pH 7.0 and 6.2, under different temperatures, in the absence or presence of added phosphate

	Temperature			
Constant	10°C	20°C	30°C	40°C
*Psoas S1*				
pH 7.0				
* K*_ADP_, μM	201 ± 34 (*n* = 2)	203 ± 13 (*n* = 4)	232 ± 29 (*n* = 2)	
* K*_ADP+Pi_, μM*	770 ± 37 (*n* = 3)	919 ± 72 (*n* = 3)	1,017 ± 52 (*n* = 3)	
* K*_Pi_, mM	16.2 ± 1.1 (*n* = 2)	28.3 ± 1.8 (*n* = 2)	31.1 ± 3.0 (*n* = 2)	41.1 ± 7.8 (*n* = 2)
* *Calc. *K*_Pi_, mM	10.6	8.5	8.9	
pH 6.2				
* K*_ADP_, μM	256 ± 32 (*n* = 2)	228 ± 36 (*n* = 2)	236 ± 29 (*n* = 2)	
* K*_ADP+Pi_, μM*	665 ± 39 (*n* = 2)	463 ± 52 (*n* = 1)	926 ± 73 (*n* = 2)	
* K*_Pi_, mM	11.5 ± 1.1 (*n* = 3)	15.6 ± 1.7 (*n* = 4)	20.5 ± 2.0 (*n* = 4)	31.1 ± 1.6 (*n* = 4)
* *Calc. *K*_Pi_, mM	18.8	23.9	10.3	
*Masseter S1*				
pH 7.0				
* K*_ADP_, μM	10.3 ± 1.2 (*n* = 2)	29.7 ± 2.8 (*n* = 2)	56.2 ± 6.5 (*n* = 2)	
* K*_ADP+Pi_, μM†	22.5 ± 2.9 (*n* = 2)	44.4 ± 4.6 (*n* = 2)	79.3 ± 6.5 (*n* = 2)	
* K*_Pi_, mM	22.3 ± 4.1 (*n* = 1)	35.0 ± 4.4 (*n* = 1)		
* *Calc. *K*_Pi_, mM	10.8	30.9	40.3	
pH 6.2				
* K*_ADP_, μM	21.8 ± 1.3 (*n* = 3)	46.8 ± 3.4 (*n* = 3)	83.9 ± 4.7 (*n* = 3)	
* K*_ADP+Pi_, μM†	52.2 ± 4.2 (*n* = 2)	82.6 ± 5.7 (*n* = 2)	174.6 ± 7.0 (*n* = 2)	
* K*_Pi_, mM	16.6 ± 0.6 (*n* = 3)	21.3 ± 0.9 (*n* = 3)	25.3 ± 1.0 (*n* = 3)	27.9 ± 1.7 (*n* = 2)
* *Calc. *K*_Pi_, mM	10.8	14.7	14.2	

Values are means ± SE. actin.S1, actin bound with S1; *K*_ADP_, dissociation constant for ADP; *K*_Pi_, dissociation constant for phosphate; *K*_ADP+Pi_, dissociation constant for ADP in the presence of phosphate.

* 30 mM P_i_. †15 mM P_i_.

The temperature dependence of the above studied biochemical steps *K*_1_*k*_+2_, *K*_ADP_, *K*_Pi_, and *K*_ADP+Pi_ data were plotted as the natural logarithm of the measured parameter against the reciprocal of temperature in degrees Kelvin (1/T °K) and fitted with linear regression using the Arrhenius (rate constants) or Van’t Hoff (equilibrium constants) equations

(7)) $$\ln K_{1}k_{+2}=\ln\left(A\right)-E_{a}/RT$$

(8)) $$\ln K_{eq}=\Delta S{}^{\circ}/R-\Delta H{}^{\circ}/RT$$

where *E*_a_ stands for activation energy, *R* is the gas constant, and *A* is a preexponential factor. The values of –*E*_a_/*R* or or ΔH°/*R* were derived from the slopes.

## RESULTS AND DISCUSSION

### ATP-Induced Dissociation Rate of Actin.S1

When actin.^Pso^S1 and actin.^Mass^S1 were mixed with ATP, as shown in Fig. 2, *A* and *B*, the observed stopped-flow transients were described by a single exponential for both myosin isoforms (Fig. 2, *A* and *B*). Keeping a fixed ATP concentration and increasing the temperature allows the best estimate of the temperature dependence of the reaction since it minimizes variation in ATP concentration between experiments. Increasing the temperature from 5 to 43°C reduced the total fluorescence signal by ~40% due to collisional quenching, but the signal change remained relatively constant, with an approximately twofold increase in fluorescence observed in all transients. The transients were therefore normalized to illustrate the change in the *k*_obs_ values. For psoas (Fig. 2*A*) and masseter (Fig. 2*B*), temperature increased the *k*_obs_ value approximately threefold in both cases over the range of measurements from 3 to 43°C. The figure shows illustrative examples of one set of transients.

Lowering the pH to 6.2 slightly increased the *k*_obs_ values for both isoforms by about 20–25% (and hence the second order rate constant *K*_1_*k*_+2_; see Table 1). Increasing temperature resulted in an average increase of threefold over the temperature range of 5–35°C. The amplitudes of the transients at pH 6.2 were again relatively stable and similar to pH 7 for ^Pso^S1 at 43%. For ^Mass^S1 the amplitudes were also stable in pH 6.2 but showed an overall increase in fluorescence from 40 to 50% of total fluorescence signal.

#### Effect of temperature.

The temperature dependence of the dissociation rate constant was examined at pH 7 and then repeated at pH 6.2 (Fig. 2, *C* and *D*). Each measurement was repeated three times, and the average values are collated in Table 2. The Arrhenius plots of the temperature dependence measurements at pH 7 and 6.2 gave well-defined straight lines over the temperature range (5–43°C). In the absence of phosphate, for psoas the activation energy (*E*_a_) values were very similar at pH 7.0 and 6.2 as shown in Fig. 2*C*, 28.3 ± 0.8 and 29.3 ± 0.8 kJ/mol, respectively. For masseter, *E*_a_ values were on average lower than the ones for fast, being pH 7.0 and 6.2, 25.7 ± 1.4 and 23.8 ± 1.1 kJ/mol, respectively (Fig. 2*D*).

**Table 2.** Thermodynamics results (E_A_ values) describing temperature dependence of the dissociation rate constant for psoas and masseter) myosin, under pH 7 and 6.2

		Pso		Mass	
Constant	± P_i_	pH 7.0	pH 6.2	pH 7.0	pH 6.2
*K*_1_*k*_+2_, kJ/mol	—	28.3 ± 0.8	29.3 ± 0.8	25.7 ± 1.4	23.8 ± 1.1
*K*_1_*k*_+2_, kJ/mol	+	37.7 ± 1.2	39.2 ± 1.6	29.9 ± 1.1	30.7 ± 0.7
*k*_−AD_, kJ/mol	—	N/A	N/A	75.9 ± 4.1	84.7 ± 6.1
*k*_−AD_, kJ/mol	+	N/A	N/A	94.4 ± 5.0	88.9 ± 3.9

Values are means ± SE. Pso, psoas; Mass, masseter; N/A, not applicable.

#### Effect of P_i_ and pH.

When the ATP-induced dissociation measurements were repeated in the presence of high phosphate concentrations, of the order that might be expected in fatigue, the observed rate constants for the dissociation reaction were twofold slower for ^Mass^S1 and two- to threefold slower for ^Pso^S1 at both pH levels compared with the data in the absence of phosphate. This is consistent with P_i_ acting as a competitive inhibitor with a *K*_i_ of 10–20 mM. It should be noted that while 30 mM P_i_ was used for ^Pso^S1, 15 mM P_i_ was used for ^Mass^S1 experiments.

The transients of both isoforms had biphasic tendencies at the low temperatures (5–10°C) at both pHs but were single exponential at all other temperatures. The origin of this additional slow phase, which had a very small amplitude (1–3%), is not known, but possible contamination by ADP was eliminated by control measurements in the presence of apyrase which converts any ADP present, which does not bind to S1, to AMP.

The amplitudes of the dissociation reaction were 50% smaller/reduced in the presence of phosphate for both, ^Pso^S1 and ^Mass^S1, indicating some loss of affinity of S1 for actin in the presence of P_i_. However, for psoas the amplitudes increased with temperature from 25 to 30% at pH 7.0 and even more dramatically from 12 to 20% at pH 6.2. This behavior was not observed with ^Mass^S1 masseter.

#### Combined effect of temperature, pH, and phosphate.

**Fig. 3.**
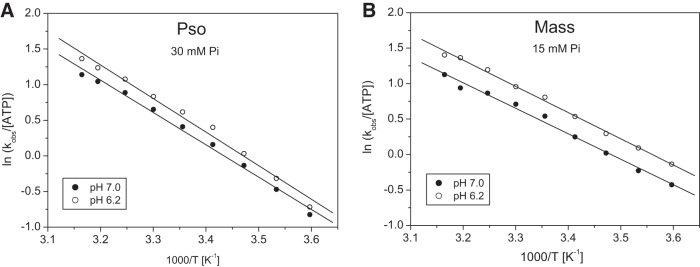
Effect of inorganic phosphate on the ATP-induced dissociation of S1 from actin, for fast (Pso) and slow (Mass) myosin isoform at pH 7.0 and 6.2, in a range of temperatures. *A*: Arrhenius plot of the *k*_obs_ of psoas at pH 7.0 and pH 6.2 in the presence of 30 mM P_i_. The linear fits (best fits superimposed) gave slopes of −4.54 ± 0.15 and −4.71 ± 0.20 K for pH 7.0 and 6.2, respectively, from which the activation energies (*E*_a_) were calculated as 37.7 ± 1.2 and 39.2 ± 1.6 kJ/mol. *B*: Arrhenius plot of the *k*_obs_ of masseter at pH 7.0 and pH 6.2 in the presence of 15 mM P_i_. The linear fits (best fits superimposed) gave slopes of −3.59 ± 0.13 and −3.694 ± 0.08 K for pH 7.0 and 6.2, respectively, from which the activation energies (*E*_a_) were calculated as 29.9 ± 1.1 and 30.7 ± 0.7 kJ/mol.

The temperature dependence of the dissociation rate constant in the presence of phosphate is shown in Fig. 3 and the activation energies determined for psoas (38 ± 1 kJ/mol) and masseter (30 ± 1 kJ/mol) were greater than in the absence of P_i_, irrespective of the pH used. Thus phosphate increased the activation energy of ^Pso^S1 at both pH values by about 10 kJ/mol, which is a larger increase than observed with masseter, where the increase was only about 5 kJ/mol in the presence of phosphate.

Rate constant of ADP release (*k*_−ADP_) was evaluated by an ADP displacement experiment, mixing actin.^Mass^S1 saturated with ADP with an excess of ATP. This measurement was not possible for ^Pso^S1 because the ADP release is too fast to measure.

**Fig. 4.**
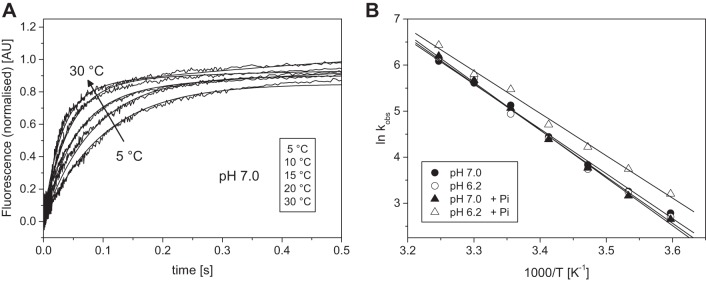
Temperature dependence of the ADP release from pyrAct.MassS1. *A*: normalized fluorescent transients observed when 0.5 µM pyrAct.MassS1 preincubated with 75 µM ADP was mixed with 8 mM ATP at different temperatures between 5 and 30°C in pH 7.0 buffer (selected transients are shown). The change in fluorescence was biphasic when observed over a time scale of 5 s; however, here only the initial fast phase is shown (fits superimposed). The *k*_obs_ for the fast phase were 16.2, 26.0, 46.3, 85.0, and 273 s^−1^ for 5, 10, 15, 20, and 30°C, respectively. *B*: Arrhenius plot of the *k*_obs_ of the ADP release rate constant of masseter at pH 7.0 and pH 6.2 in the absence and presence of 15 mM P_i_. The linear fits (best fits superimposed) gave slopes of −9.72 ± 0.24 and −10.09 ± 0.33 K for pH 7.0 and 6.2, and −10.36 ± 0.23 and −9.20 ± 0.31 K for pH 7.0 + P_i_ and pH 6.2 + P_i_, respectively. The activation energies (*E*_a_) were calculated as 75.9 ± 4.1 and 84.7 ± 6.1 kJ/mol for pH 7.0 and 6.2 without phosphate, and 94.4 ± 5.0 and 88.9 ± 3.9 kJ/mol for pH 7.0 and pH 6.2, respectively, in the presences of phosphate.

Displacement of ADP from actin.^Mass^S1 by a large excess of ATP was biphasic. The transients were well defined with stable amplitudes of 24 and 6% for the fast and slow phase, respectively (as shown in Fig. 4*A*). These amplitudes were similar under all conditions explored. The fast phase defines the rate constant at which ADP is released and is thought to limit the velocity of shortening of a masseter muscle (4). The slower phase is an off pathway event and will not be considered further here. The *k*_obs_ of the ADP release was 85 s^−1^ at 20°C (pH 7.0) and compares well to published results of 94 s^−1^ by Bloemink et al. (4).

The reaction was measured over the temperature range of 5–30°C at pH 6.2 and 7.0, and in the presence of 15 mM P_i_. The *k*_obs_ values are summarized in the Arrhenius plot in Fig. 4*B*. The *k*_obs_ values increased from 16.2 at 5°C to 273 s^−1^ at 30°C with similar values at pH 7.0 and pH 6.2 throughout the temperature range used. Above 30°C the reaction was too fast to measure reliably. Thus the activation energy was large with similar values at both pH levels studied.

The addition of 15 mM P_i_ had little effect at pH 7.0. At pH 6.2, however, we saw a 30–50% increase in *k*_obs_ in the presence of phosphate and a small change in the activation energy.

### ADP Dissociation Constant (K_ADP_)

The ADP dissociation constant (*K*_ADP_) for pyr.actin.S1 was measured by the competitive inhibitor approach as described in materials and methods.

**Fig. 5.**
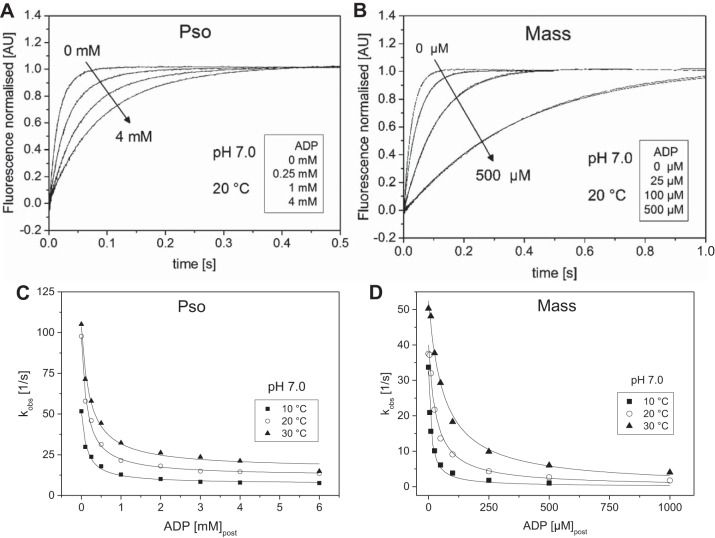
Temperature dependence of the ADP dissociation constant (*K*_ADP_) for fast (Pso) and slow (Mass) A.S1 at pH 7.0. *A*: normalized fluorescent transients observed when 0.5 µM pyrAct.PsoS1 was mixed with 25 µM ATP with various concentrations of ADP present at 20°C in pH 7.0 buffer. The change in fluorescence was fitted by a single exponential equation (best fits superimposed). The *k*_obs_ determined were 87.7, 45.9, 21.4, and 14.5 s^−1^ for zero, 0.25, 1, and 4 mM ADP, respectively, with an amplitude of 30% of total fluorescence. *B*: fluorescent transients observed when 0.5 µM pyrAct.MassS1 was mixed with 25 µM ATP with various concentrations of ADP present at 20°C in pH 7.0 buffer. The change in fluorescence was fitted to a single exponential equation (best fits superimposed). The *k*_obs_ determined were 37.5, 21.7, 9.1, and 2.6 s^−1^ for zero, 25, 100, and 500 µM ADP, respectively, with an amplitude of 30% of total fluorescence. *C*: plot of the observed rate constants as a function of [ADP] for psoas in pH 7.0 buffer at 10, 20. and 30°C. The data sets were fitted to a hyperbole to obtain the ADP dissociation constant (*K*_ADP_) for each temperature: 131 ± 16 µM (10°C), 140 ± 14 µM (20°C), and 213 ± 29 µM (30°C) for the depicted data. Refer to Table 1 for average values from measurements in different days. *D*: plot of the observed rate constants as a function of [ADP] for masseter in pH 7.0 buffer at 10, 20, and 30°C. The data sets were fitted to a hyperbole to obtain the ADP dissociation constant (*K*_ADP_) for each temperature: 9.6 ± 0.7 µM (10°C), 31.3 ± 4.0 µM (20°C), and 62.4 ± 6.1 µM (30°C) for the depicted data. Refer to Table 1 for average values from measurements in different days.

ADP included in the ATP solution competes with ATP for binding to the pyr.actin.S1 and slows the *k*_obs_ value as shown in Fig. 5. The ADP dissociation constant was 168 µM for ^Pso^S1 and 31 µM for ^Mass^S1 at 20°C and pH 7.0, as reported previously (22). This large difference in the affinity of actin.S1 for ADP is a major characteristic of a fast vs. a slow myosin isoform. As reported previously, the ADP affinity for psoas actin.S1 was relatively unaffected by temperature (about 200 ± 30 µM between 10 and 30°C) while for masseter the effect was much greater, with the affinity becoming weaker by approximately sixfold from 9.6 µM at 10°C to 62.4 at 30°C, at pH 7.0.

#### Effect of pH.

A change in pH did not affect the ADP affinity for psoas (Table 1) over the temperature range studied (also Fig. 5*C*). Lowering the pH to 6.2 with ^Mass^S1 resulted in twofold weaker *K*_ADP_ values than at pH 7.0 (from 10 to 22 µM at 10°C). However, this effect of pH was not as pronounced at higher temperatures (only weakening by 1.5-fold at 30°C; see Table 1).

### Phosphate Dissociation Constant (K_Pi_)

**Fig. 6.**
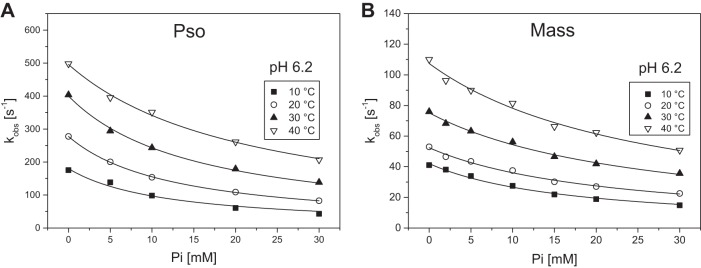
Phosphate (P_i_) dissociation constant for actomyosin complex (AM) in the absence of ADP (phosphate) at pH 6.2, for fast (Pso) and slow (Mass) myosin isoform. *A*: plot of the observed rate constants of the ATP-induced dissociation of 0.5 µM pyrAct.S1 by 50 µM ATP as a function of [P_i_] for psoas (pH 6.2 buffer) at 10 to 40°C. The data sets were fitted to a hyperbole to obtain the P_i_ dissociation constant (*K*_Pi_) for each temperature: 11.7 ± 1.8 mM (10°C), 12.7 ± 0.2 mM (20°C), 15.5 ± 0.7 mM (30°C), and 22.1 ± 1.2 mM (40°C). Refer to Table 1 for average values for from measurements in different days. *B*: plot of the observed rate constants of the ATP-induced dissociation of 0.5 µM pyrAct.S1 by 25 µM ATP as a function of [P_i_] for masseter (pH 6.2 buffer) at 10 to 40°C. The data sets were fitted to a hyperbole to obtain the P_i_ dissociation constant (*K*_Pi_) for each temperature: 17.3 ± 1.1 mM (10°C), 22.0 ± 1.4 mM (20°C), 26.1 ± 1.3 mM (30°C), and 26.7 ± 2.1 mM (40°C). Refer to Table 1 for average values from measurements in different days.

The dissociation constant of P_i_ for actin.S1 (*K*_Pi_) was measured but the range of P_i_ concentrations accessible was restricted by the need to maintain a constant ionic strength. As P_i_ was increased, the concentration of KCl in the buffer was decreased and the maximum phosphate concentration used was 30 mM. Figure 6 shows the plots of *k*_obs_ as a function of phosphate concentration for the two myosin isoforms. These show the expected inhibition as [P_i_] is increased, with an average *K*_Pi_ value of 15 mM at 10°C decreasing to 41 mM at 40°C for actin.^Pso^S1 at pH 7.0. Decreasing the pH to 6.2 did not significantly affect the *K*_Pi_ values for ^Pso^S1 (11 mM at 10°C, decreasing to 32 mM at 40°C; see also Table 1).

Repeating the measurements with ^Mass^S1 gave a *K*_Pi_ of 22 mM at 10°C, weakened to 35 mM at 20°C (pH 7.0). Lowering the pH to 6.2 resulted in an average *K*_Pi_ value of 17 mM at 10°C, weakening to 28 mM at 40°C. Thus a differential response of slow myosin to P_i_ was observed with temperature, with the slow myosin while starting off less sensitive to P_i_ at 10°C becoming more sensitive to P_i_ at 40°C.

**Fig. 7.**
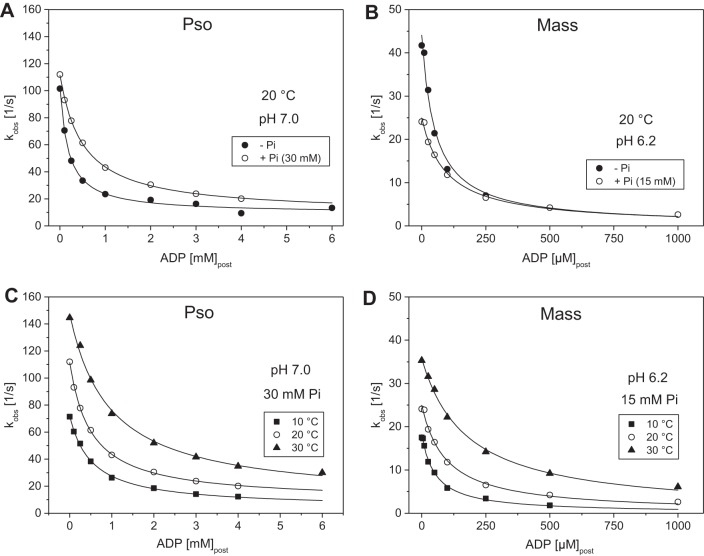
Effect of phosphate (P_i_) on the *K*_AD_ of fast (Pso) and slow (Mass) A.S1. *A*: plot of the observed rate constants as a function of [ADP] for psoas in the presence and absence of added 30 mM P_i_ (pH 7.0 buffer at 20°C). The data sets were fitted to a hyperbole to obtain the ADP dissociation constant (*K*_ADP_) ± P_i_: 175 ± 22 µM (no P_i_) and 510 ± 22 µM (with P_i_). Refer to Table 1 for average values from measurements in different days. *B*: plot of the observed rate constants as a function of [ADP] for masseter in the presence and absence of added 15 mM P_i_ (pH 6.2 buffer at 20°C). The data sets were fitted to a hyperbole to obtain the ADP dissociation constant (*K*_ADP_) ± P_i_: 48.4 ± 6.8 µM (no P_i_) and 94.5 ± 8.1 µM (with P_i_). Refer to Table 1 for average values for from measurements in different days. *C*: plot of the observed rate constants as a function of [ADP] for psoas in pH 7.0 buffer in the presence of added 30 mM P_i_ at 10, 20, and 30°C. The data sets were fitted to a hyperbole to obtain the ADP dissociation constant (*K*_ADP_) for each temperature: 530 ± 36 µM (10°C), 510 ± 22 µM (20°C), and 942 ± 117 µM (30°C). Refer to Table 1 for average values from measurements in different days. *D*: plot of the observed rate constants as a function of [ADP] for masseter in pH 6.2 buffer in the presence of added 15 mM P_i_ at 10, 20, and 30°C. The data sets were fitted to a hyperbole to obtain the ADP dissociation constant (*K*_ADP_) for each temperature: 52.2 ± 5.8 µM (10°C), 94.5 ± 8.1 µM (20°C), and 175.3 ± 9.7 µM (30°C). Refer to Table 1 for average values for from measurements in different days.

ADP dissociation constant in the presence of phosphate (*K*_ADP+Pi_) was evaluated as for the ADP dissociation constant but using fixed amounts of inorganic phosphate (30 mM in the case of ^Pso^S1 and 15 mM with ^Mass^S1). The presence of 30 mM P_i_ weakened the ADP dissociation constant (*K*_ADP+Pi_) for actin.^Pso^S1 three- to fourfold [from about 170 µM to 890 µM at 20°C (pH 7.0)] as shown in Fig. 7*A* and Table 1. Repeating the measurement at different temperatures showed the apparent *K*_ADP_ weakening from around 500 µM at 10–20°C to 942 µM at 30°C (Fig. 7*C* and Table 1). For masseter the effects of P_i_ were less marked, with the *K*_ADP_ weakening only one- to twofold across the temperature range at pH 7.0. Overall, it appears that phosphate competes with ADP binding to fast A.M, but has little effect on ADP binding in slow A.M. The formation of an A.M.ADP.Pi complex (see *Data Fitting and Interpretation Approach*) is not supported under our experimental conditions.

Lowering the pH to 6.2 resulted in a smaller effect of phosphate on the ADP dissociation constant for actin.^Pso^S1, changing only twofold from 228 to 514 µM at 20°C (compared with the three- to fourfold change seen at pH 7.0). This reduced effect of phosphate was seen across the temperature range used. In actin.^Mass^S1, 15 mM P_i_ weakened the ADP affinity twofold from 47 to 94 µM at 20°C, and a similar twofold weakening of the *K*_ADP_ in phosphate (*K*_ADP+Pi_) was seen at the other temperatures used at pH 6.2.

### Apparent Phosphate Dissociation Constant (K_Pi app_)

The apparent dissociation constant of phosphate for acto-myosinS1 (*K*_Pi app_) in the presence of ADP was calculated from the ADP dissociation constants measured in the absence (*K*_ADP_) and presence of phosphate (*K*_ADP+Pi_) as detailed in the methods. At pH 7.0 and low temperature the *K*_Pi app_ of actin.^Pso^S1 was similar to the *K*_Pi_ value measured (11 mM and 16 mM, respectively, at 10°C). At higher temperatures the *K*_Pi_ of actin.^Pso^S1 was weakened to 30–40 mM, the *K*_Pi app_, however, remained at about 10 mM for the whole temperature range used.

At pH 6.2 the *K*_Pi_ of psoas was 30% tighter than at pH 7.0 but otherwise showed the same behavior as temperature was increased (weakening from 15 mM at 10°C to 32 mM at 40°C). The *K*_Pi app_, however, appears twofold weaker at pH 6.2 for psoas with 24 mM and tightens to about 16 mM as temperature is increased.

For actin.^Mass^S1, we observed a different behavior of the apparent phosphate dissociation constant; while the measured *K*_Pi_ values at pH 7.0 were similar to psoas across the temperature range used, the *K*_Pi app_ showed distinct temperature dependence, weakening from 10 to 40 mM with temperature. The *K*_Pi_ values of masseter were unaffected by a change in pH to 6.2 and remained similar to psoas at 22 and 35 mM (10 and 20°C, respectively). The *K*_Pi app_, however, lost its temperature dependence when the pH was lowered to 6.2 and the value remained relatively unaffected at 10–15 mM for actin.^Mass^S1 throughout the temperature range used.

#### Relevance to working muscle.

Work by us and others indicated an important role for P_i_ in tension generation as conditions that affect actomyosin affinity would affect, in proportion, force generation. With the assumption that A.M force-generating states are in an effective equilibrium with the non-force-generating states at the beginning of the working stroke, past skinned psoas fiber work suggested that, with increasing [P_i_] the free energy of the states that precede P_i_ release decrease as −RT ln[P_i_] (from the slope of the force-ln[P_i_] relationship, relative to the free energy of states after P_i_ release, leading to progressive depopulation of the force-generating states and thus reducing tension generation (33). Earlier observations by Tesi et al. (54) highlighted differences between slow and fast myofibrils in tension response to phosphate, with indications of stronger actomyosin bonds in slow muscle. The combination of low pH and high P_i_ was shown to synergistically inhibit velocity of contraction in skinned fibers (36, 43) adding further support to the notion that in fatigue conditions, the combined effect of P_i_ and protons on muscle performance would come about either by decreasing the force per bridge and/or increasing the number of low-force bridges. These and other studies indicated that the effect of P_i_ on its own is moderate at higher temperatures but in combination with low pH it can substantially affect muscle power by affecting actomyosin interaction. The present work adds important information to explain how P_i_’s interaction changes the ADP dissociation constant for AM and ultimately ATP-induced dissociation of AM, thus the speed of the cross-bridge cycle.

### Concluding Remarks

The phenomena we studied are at a lower level of component configuration, actin, and myosin S1 in solution. We cannot therefore account for myosin cooperativity and coordinated responses to load, which could affect the hypothesized limiting processes. While experimental data imply such cooperativities (3), emerging behaviors are difficult to assess and model, a situation further complicated by the difficulty of incorporating intrahead actions into models (40). At the macroscopic level, many studies have examined fatigue effects on mechanical function using single fibers (most however at nonphysiological temperature); there are also isolated muscle and whole limb investigations (however with no control over metabolites levels); all these macroscopic studies have theorized about what may be occurring at the molecular level. Fewer studies have attempted a “molecular explanation” of how velocity is affected in muscle fatigue [e.g., using in vitro motility (16)]. Ours is the first study to employ solution transient kinetics to study how key fatigue factors affect the ATP-induced dissociation step of fast and slow S1 from actin (a critical part of the cycle that affects overall velocity). More information of the other events in the cycle, and the temperature dependence of these events for both fiber types, is needed to support future modeling attempts.

It remains to be seen how our findings can be integrated at the higher level “behavior” of large myosin ensembles interacting with actin filaments, outside or inside an organized sarcomere. It is expected that in such situations other laws may apply when the myosin type effect on contractile behavior is further modulated depending on interactions with intracellular factors and overall muscle action regulation.

We expect that, given the undisputed phenotypic effect of myosin types as observed in mammalian physiology, our data provide highly relevant insights into the mechanochemical coupling factors that distinguish the fiber types. Phosphate dependence of ATP-induced dissociation is modulated by variations in actin affinity. Such variations could help modulate the phosphate dependence of force and velocity, and may explain why phosphate sensitivity appears to be in part temperature- and muscle type-dependent.

## GRANTS

The authors acknowledge support from various sources as follows: M. A. Geeves was supported by the British Heart Foundation Grant PG30200. Also, C. Karatzaferi and M. A. Geeves have received cofinancing by the European Union (European Social Fund – ESF) and Greek national funds through the Operational Program “Educational and Lifelong Learning” of the National Strategic Reference Framework (NSRF) – Research Funding Program: Thales (MuscleFun Project-MIS 377260) Investing in knowledge society through the European Social Fund. Moreover, C. Karatzaferi has received funding from the European Union’s Horizon 2020 research and innovation program under the Marie Skłodowska-Curie grant agreement No 645648, “Muscle Stress Relief” and support by the COST Action CM1306 “Understanding Movement and Mechanism in Molecular Machines.”

## DISCLOSURES

No conflicts of interest, financial or otherwise, are declared by the authors.

## AUTHOR CONTRIBUTIONS

C.K. and M.A.G. conceived and designed research; C.K., N.A., and M.A.G. performed experiments; C.K., N.A., and M.A.G. analyzed data; C.K., N.A., and M.A.G. interpreted results of experiments; C.K. and N.A. drafted manuscript; C.K. and M.A.G. edited and revised manuscript; C.K., N.A., and M.A.G. approved final version of manuscript; N.A. prepared figures.
